# Colorectal cancer survival among Ministry of National Guard-Health Affairs (MNG-HA) population 2009–2017: retrospective study

**DOI:** 10.1186/s12885-021-08705-8

**Published:** 2021-08-25

**Authors:** Mesnad Alyabsi, Fouad Sabatin, Majed Ramadan, Abdul Rahman Jazieh

**Affiliations:** 1 Population Health Research Department, King Abdullah International Medical Research Center, P.O. Box 22490, Riyadh, 11426 Saudi Arabia; 2 King Saud bin Abdulaziz University for Health Sciences, P.O. Box 22490, Riyadh, 11426 Saudi Arabia; 3 Oncology Department, Ministry of National Guard - Health Affairs, Riyadh, Saudi Arabia

**Keywords:** Colorectal cancer (CRC), Registry, SEER, Survival, Saudi Arabia

## Abstract

**Background:**

Colorectal cancer (CRC) is the most diagnosed cancer among males and third among females in Saudi Arabia, with up to two-third diagnosed at advanced stage. The objective of our study was to estimate CRC survival and determine prognostic factors.

**Methods:**

Ministry of National Guard- Health Affairs (MNG-HA) registry data was utilized to identify patients diagnosed with CRC between 2009 and 2017. Cases were followed until December 30th, 2017 to assess their one-, three-, and five-year CRC-specific survivals. Kaplan-Meier method and Cox proportional hazard models were used to assess survival from CRC.

**Results:**

A total of 1012 CRC patients were diagnosed during 2009–2017. Nearly, one-fourth of the patients presented with rectal tumor, 42.89% with left colon and 33.41% of the cases were diagnosed at distant metastasis stage. The overall one-, three-, and five-year survival were 83, 65 and 52.0%, respectively. The five-year survival was 79.85% for localized stage, 63.25% for regional stage and 20.31% for distant metastasis. Multivariate analyses showed that age, diagnosis period, stage, nationality, basis of diagnosis, morphology and location of tumor were associated with survival.

**Conclusions:**

Findings reveal poor survival compared to Surveillance, Epidemiology, and End Results (SEER) population. Diagnoses at late stage and no surgical and/or perioperative chemotherapy were associated with increased risk of death. Population-based screening in this population should be considered.

## Introduction

Worldwide, colorectal cancer (CRC) is the third most common cancer with more than 1.9 million new cases were diagnosed in 2020 [1]. Age standardized incidence rates are highest in developed countries such as Australia, New Zealand, Europe and North America due to urbanization and high-calorie diet; whereas incidence rates are lowest in Africa and developing Asian countries and the Gulf Cooperation Council (GCC) [1]. Although incidence rates have been declining in Western countries, due to systematic screening programs which aim to detect and remove precancerous polyps, the rates have been increasing considerably in GCC countries. This is probably due to increasing prevalence of CRC risk factors such as lack of physical activities, smoking and unhealthy diet, along with the lack of organized screening programs.

More than 935,000 CRC deaths were reported globally in 2020 [1]. Unlike developed countries where mortality rates have been decreasing, the rates are increasing in developing countries. The declining mortality rates in developed countries pertain to a combination of early detection efforts, where CRC is diagnosed at early curable stage, and effective treatments. The increasing mortality rates in developing countries that coincide with increasing incidence reflects increased prevalence of CRC risk factors, lack of screening at the population level, and treatment of cases most likely presented with advance diseases [1–4].

In Saudi Arabia, CRC is the most common cancer in Saudi males and the third among Saudi females [5, 6], with an age standardized incidence rate tripled since the establishment of cancer registry in 1994 [6, 7]. In 2017, the age standardized rates per 100,000 were 10.6 for males and 8.2 for females, with highest rates reported in Riyadh region [6]. The increasing incidence rate is the highest among the GCC countries because of the increasing adoption of sedentary lifestyle, smoking and Western cuisine [4]. Despite a well-established healthcare system, and the initiating of national CRC screening guidelines, up to 73% of CRC tumors diagnosed at late stage, a reflection of lack of early CRC detection programs [3, 5, 6].

The World Health Organization (WHO) estimated mortality rates in 2016 to be 10 per 100,000 among Saudi males and 7 per 100,000 among females [8]. While mortality data are unavailable from Saudi Arabia, prior studies examined CRC survival among Saudi population. For example, two studies investigated the overall survival between 1994 and 2004 and showed that the 5-year CRC survival was 44.6% [9, 10]. Another prospective study found that the median survival time among CRC patient to be 54 months [11]. However, previous research lack important prognostic factors (e.g., tumor morphology, basis of diagnosis and marital status) [10], or failed to report survival after adjustments of prognostic factors [9, 11].

Numerous factors influence and predict the outcome of CRC. Among studied factors are: age, socioeconomic (SES), comorbidities, tumor characteristics such as location, stage at diagnosis and degree of differentiation and treatment [12]. For instance, tumors diagnosed at early stage and those that are well-differentiated have better survival than their counterparts. Likewise, tumors with no lymphovascular invasion or distant metastases have better prognosis than those with invasion or metastases.

In view of that, current study was designed to elucidate factors associated with CRC survival among MNG-HA population. The findings of this study are intended to bring importance to the most diagnosed cancer in Saudi males. Hence, interventions may be planned, and policy devised to provide high-quality cancer care and detect disease when survival are highest.

## Methods

### Data sources

The current study is a retrospective cohort study using data from the (MNG-HA) Cancer Registry. The registry, which was established in 1994, collect information about cancer patients’ demographic information, clinical characteristics including type of cancers, their location and extent at the time of diagnosis. The registry captures all cases diagnosed and treated at King Abdulaziz Medical City (KAMC) in Riyadh.

Follow-up information is captured in the data at each visit including patients’ last contact date. Information on vital status is obtained annually through contacting patients or next of kin, and date and cause of death if dead are obtained from next of kin.

### Study population

#### Identification of patients

All individuals eligible for analysis were aged ≥18 years diagnosed with a first primary invasive, malignant colorectal cancer International Classification of Diseases (ICD) (ICD-10 C18–C20) between 2009 and 2017, who were resident in Saudi Arabia and registered in MNG-HA hospitals system with follow-up time started at the date of CRC diagnosis in the registry and ended on the date of death, date of last contact or when study ended on December 31, 2017 whichever came first. The MNG-HA population include military service personnel and their dependents, members of civilian workforce and students from the MNG-HA related healthcare system. The population (> 328,000 individuals) is served by tertiary care hospitals and four main primary and secondary care clinics. The oncology center at KAMC is a major oncology center in Riyadh region with estimated population of 8.2 million individuals. Approximately 30% of all cancer cases in Saudi Arabia were treated at KAMC oncology center in Riyadh. There were 1017 individuals diagnosed with CRC between years 2009–2017. Of 1017 eligible patients, 5 (0.5%) were excluded due to unknown survival status, admission or contact dates.

### Study variables

#### Patient and tumor characteristics

Patient demographics including age at diagnosis, gender, marital status and nationality were all extracted from electronic health records. Clinical variables such as tumor’s topography, morphology, treatment (surgery, radiotherapy and/or chemotherapy), grade, extent and basis of diagnosis, were derived from pathology reports and/or surgical specimens and were coded using (ICD-10 C18–C20).

The anatomic tumor locations were categorized according to the ICD for Oncology-third edition topography as follow: right colon (i.e., cecum, ascending colon, hepatic flexure of colon and transverse colon), left colon (i.e., splenic flexure of colon, descending colon, and sigmoid), colon not otherwise specified (NOS) and rectum (rectosigmoid junction and rectum) [13–15].

SEER Summary Staging of localized, regional, and distant groups was used in this study [16].

#### Outcome variable

CRC-specific survivals were determined from registry records of survival time and the ascertainment of vital status. Follow-up time started at the date of CRC diagnosis in the registry and ended on the date of death, date of last contact or when study ended on December 31, 2017 whichever came first. To allow for 5-years of survival estimates, we restricted the population to patients diagnosed with CRC anytime up to December 31, 2017. We excluded patients with follow-up of < 1 months [17]. Completeness of follow-up was computed at each time interval using Clark’s Completeness Index (CCI) as well as a simplified person-time (SPT) method [17–19]. The resulting CCI and SPT were 72.38 and 80.64%, respectively.

### Statistical analysis

The demographic and clinical characteristics were assessed across three periods (2009–2011, 2012–2014 and 2015–2017) using Cochran-Armitage or Cochran-Mantel-Haenszel tests, where appropriate. The 1-, 3- and 5-year CRC-specific survival were estimated using Kaplan-Meier product limit method and differences between curves were assessed using log-rank test. The data was right-censored, there was no patient censored alive before the end of the complete follow-up time, thus all censored subjects were due to death only. Cox proportional hazards regression models were used to assess the univariate and multivariate association between CRC-specific survival time and covariates. We assessed the proportional hazard assumption using Schoenfeld residuals. Variables included in the multivariate regression model were diagnosis period, age, gender, marital status, nationality, stage at diagnosis, tumor’s topography, morphology, grade, surgery, chemotherapy, radiotherapy, and basis of diagnosis (histology of primary vs. metastasis). We assessed interaction between all covariates and found a significant interaction between diagnosis period and basis of diagnosis at the univariate analysis, but the interaction was insignificant when added to the multivariate model and was dropped from the final model. Backward elimination was used during multivariate analysis to retain all variables with *P* < 0.20.

All statistical tests were 2-sided, and findings were considered statistically significant at *P* < .05. All analyses were conducted using SAS statistical software version 9.4 (SAS Institute Inc. Cary, NC).

## Results

The application of the eligibility criteria resulted in a cohort of 1012 patients diagnosed with CRC between 2009 and 2017. Tables 1, and 2 show the demographic and clinical characteristics of the study population. Overall, there has been a significant increase in the diagnosis of CRC between 2009 and 2011 28.46% and 2012–2014 35.47%, however this increase has declined in 2015–2017 32.51%. There was 57.51% men, 74.51% married individuals and 92.49% Saudis diagnosed with CRC. Single individuals represent 5.4% of the CRC population. While 42.89% of colon cancer patients were diagnosed with left-side tumor, about one-fourth of CRC patients were diagnosed with rectal tumor and 83.1% presented with stage III or IV tumors. The most diagnosed tumors were a histology of adenocanrcinoma of primary tumor that were moderately differentiated.

**Table 1:** Demographic and Clinical Characteristics by Diagnosis Period, MNGHA 2009-2017^a^

	Total		Diagnosis period						
**Characteristics**	**N**^**d**^	**%, SD**	**2009–2011**		**2012–2014**		**2015–2017**		*P*
Total	1012	100	288	28.46	395	35.47	329	32.51	
**Age (years)**									0.0012^b^
Mean (SD)	68.03	14.39	69.65	14.31	69.19	14.22	65.21	14.31	
< =40	40	3.95	8	2.78	14	3.54	18	5.47	
41–50	68	6.72	13	4.51	20	5.06	35	10.64	
51–60	186	18.38	48	16.67	72	18.23	66	20.06	
61–70	288	28.46	92	31.94	102	25.82	94	28.57	
71–80	237	23.42	60	20.83	105	26.58	72	21.88	
> =81	193	19.07	67	23.26	82	20.76	44	13.37	
**Gender**									
Female	430	42.49	127	40.51	161	40.76	142	43.16	0.65
Male	582	57.51	161	59.49	234	59.24	187	56.83	
**Marital status**									0.44
Single	55	5.43	15	5.21	23	5.82	17	5.17	
Married	748	74.91	224	77.78	294	74.43	240	72.95	
Widowed/divorced	57	5.63	15	5.21	17	4.30	25	7.60	
Unknown	142	14.03	34	11.89	61	15.44	47	14.29	
**Nationality**									0.36
Non-Saudi	76	7.51	21	2.08	35	3.46	20	1.98	
Saudi	936	92.49	267	97.92	360	96.54	309	98.02	
**Tumor Site**									0.002
Right colon	184	18.18	42	14.58	67	16.96	75	22.79	
Left colon	434	42.89	121	42.01	174	44.05	139	42.25	
Colon-non specified	160	15.18	62	21.53	65	16.46	33	10.03	
Rectum	234	23.12	63	21.87	89	22.53	82	24.92	
**Tumor Morphology**									0.78^c^
Adenocarcinoma (AC), NOS	854	84.39	238	82.64	339	85.82	277	84.19	
Mucinous AC	63	6.23	18	6.25	23	5.82	22	6.69	
Mucin-producing AC	12	1.19	4	1.39	2	0.51	6	1.82	
Signet ring cell carcinoma	14	1.38	5	1.74	7	1.77	2	0.61	
AC in villous/tubuvillous adenoma	16	1.58	6	2.08	5	1.27	5	1.52	
Others	53	5.34	17	5.90	19	4.81	17	5.17	
**Tumor grade**									0.48
Well differentiated	33	3.26	12	4.17	9	2.28	12	3.65	
Moderately differentiated	774	76.48	222	77.08	296	74.94	256	77.81	
Poorly differentiated/Anaplastic	67	6.62	15	5.21	29	7.34	23	6.99	
Unknown	138								
**Stage at diagnosis**									0.0002
Localized	227	22.43	72	18.23	111	28.10	44	13.37	
Regional	441	40.61	111	37.63	146	36.96	154	46.81	
Distant metastasis	338	33.41	96	33.33	127	32.15	115	34.95	
Unknown	36	3.56	9	3.13	11	2.78	16	4.86	
**Basis of diagnosis**									0.02^c^
Histology of primary	972	96.05	275	95.49	381	96.46	316	96.04	
Histology of metastases	28	2.77	8	2.78	9	2.28	11	3.34	
Others	12	1.19	5	1.74	5	1.27	2	0.61	
									0.65
**Surgery**									
Yes	426	42.09	179	62.15	157	39.75	90	27.36	
No	586	57.91	109	37.85	238	60.25	239	72,64	
**Chemotherapy**									< 0.0001
Yes	411	40.61	180	62.5	172	43.54	79	24.01	
No	601	59.39	108	37.5	243	56.46	250	75.99	
**Radiotherapy**									0.007
Yes	95	9.39	40	13.89	32	8.10	23	6.99	
No	917	90.61	248	86.11	363	91.99	306	93.01	

^a^ Data represent colorectal cancer patients registered in MNG-HA hospitals system between 1 January 2009 and 31 December 2017

^b^*P*-values refer to comparisons between years range using Chi-square test

^c^ P-values refer to comparisons between marital status groups using Fisher exact test

^d^ “N” total sample size

**Table 2:** CRC-specific Survival by Patients’ Demographic Characteristics (2009–2017)

	1-year			3-year		5-year	
Overall	N	%	(95% CI)	%	(95% CI)	%	(95% CI)
	1012	83.09	(80.44, 85.41)	65.0	(61.39, 65.77)	52.0	(46.35, 57.24)
Diagnosis period^*^							
2009–2011	288	83.76	(78.79, 87.66)	65.17	(59.88, 72.16)	51.89	(44.44, 58.82)
2012–2014	395	81.16	(76.61, 84.91)	62.91	(54.87, 69.91)	–	–
2015–2017	329	84.08	(79.01, 88.02)	65.16	(48.59,77.56)	–	–
**Age**							
≤ 40	40	77.28	(59.33, 88.05)	61.47	(37.19, 78.71)	30.73	(1.91, 70.36)
41–50	68	94.82	(84.75, 98.31)	65.77	(46.36, 79.59)	50.74	(27.00, 73.25)
51–60	186	86.71	(80.26, 91.17)	68.57	(59.45, 80.44)	60.28	(43.75, 73.36)
61–70	288	89.87	(85.98, 93.96)	69.61	(61.35, 76.44)	63.41	(53.65, 71.64)
71–80	237	79.27	(73.00, 84.24)	67.17	(58.72, 74.26)	46.17	(33.91, 57.59)
≥ 81	193	67.66	(59.83, 74.30)	47.40	(37.27, 56.85)	35.91	(24.37, 57.56)
**Gender**							
Female	430	82.26	(78.05, 84.93)	61.34	(54.80, 67.03)	51.14	(43.01, 58.68)
Male	582	83.73	(80.16, 86.71)	67.83	(60.01, 71.57)	53.38	(45.32, 67.78)
Marital status^*^							
Single	55	90.58	(76.57, 96.40)	61.96	(31.07, 82.19)	24.78	(4.10, 54.37)
Married	748	82.35	(79.23, 85.05)	65.43	(60.56, 68.53)	53.97	(47.77, 59.76)
Widowed/divorced	57	80.54	(64.61, 89.83)	47.13	26.23, 65.51)	–	–
Unknown	142	82.40	(74.55, 88.02)	63.82	(48.89, 75.43)	58.50	(41.19, 72.32)
Nationality^*^							
Saudi	936	82.52	(79.73, 84.96)	65.01	(60.61, 67.89)	52.54	(46.77, 57.98)
Non-Saudi	76	88.31	(76.39, 94.42)	61.28	(37.06, 78.53)	–	–

* Indicate no pateints alive to calcuate survival rate for survival year period

* Indicate no pateints alive to calcuate survival rate for survival year period

By the end of follow-up, 275 patients censored due to death, 274 deaths were due to CRC and 1 was due to other causes. No patient was censored alive before the end of the complete follow-up time. The 1-, 3- and 5-year CRC survival were 83.09 (95% CI: 80.44, 85.41), 65% (95% CI: 61.39, 65.77) and 52% (95% CI: 46.35, 57.24), respectively (Table 2). Male, single individuals and those who are non-Saudi have higher 1-year survival than their counterparts, while married and Saudis have higher 3 and 5-years survival than their counterparts. The 1-year survival has improved slightly during the latest period (2015–2017) compared to prior periods (Table 2).

**Fig. 1:**
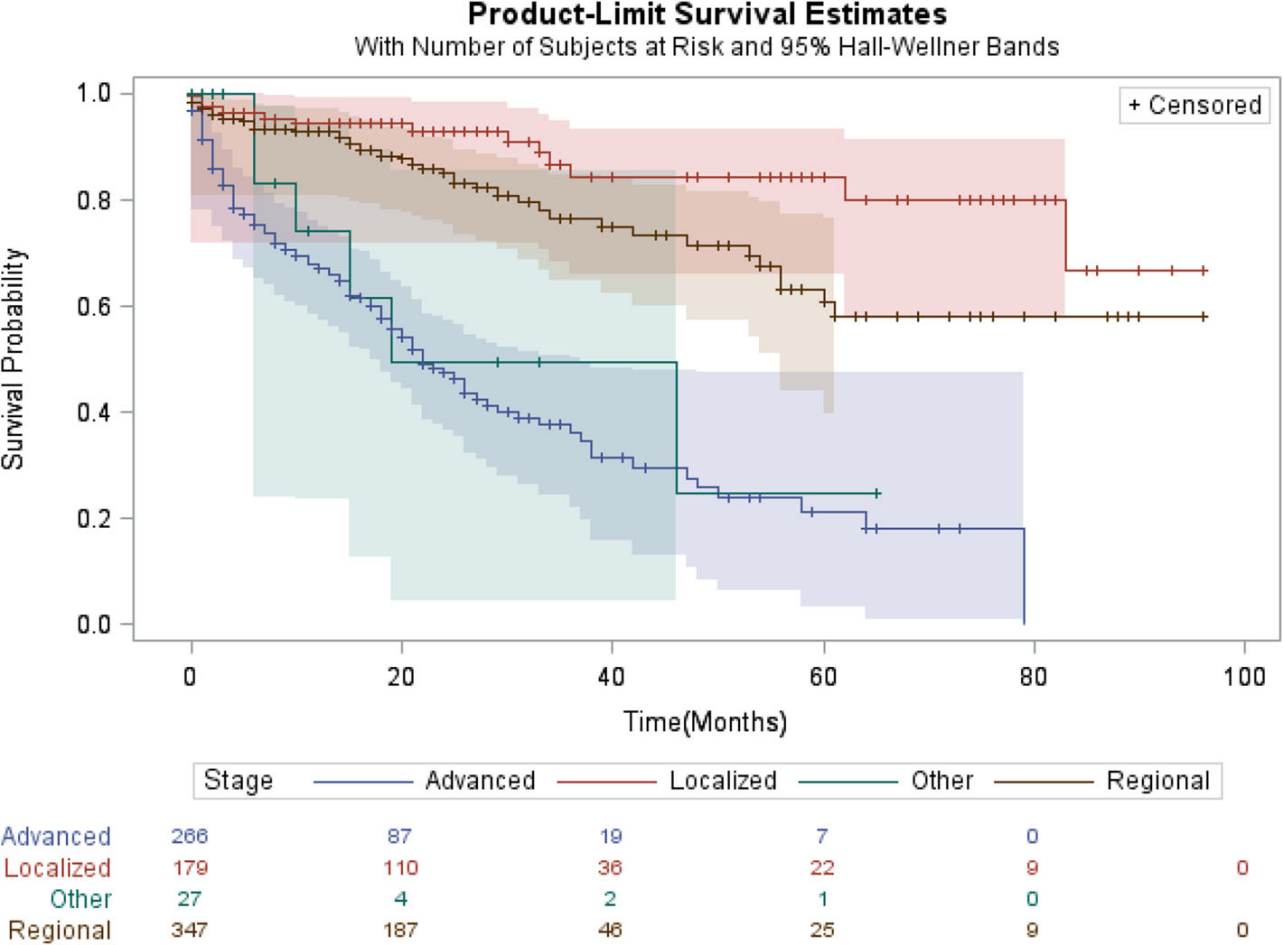
Kaplan-Meier Survival Rate by Stage

According to patient’s clinical characteristics, patients with a tumor located at left colon, those with grade moderately differentiated tumors or patients presented with localized tumor of a primary origin and adenocarcinoma in nature were more likely to have higher survival (Table 3). Survival decreases significantly with the increased stage at diagnosis (the 5-year survival of localized, regional and distant metastatic tumors were 79.85, 63.25 and 20.31%, respectively) (Table 3, and Fig. 1). Patients who have been treated with surgery and perioperative chemotherapy have significantly higher survival relative to those who did not treat with it (Table 3).

**Table 3:** CRC-specific Survival by Patients’ Clinical Characteristics (2009–2017)

	1-year			3-year		5-year	
Overall	N	%	(95% CI)	%	(95% CI)	%	(95% CI)
**Tumor Site**							
Right colon	184	81.16	(73.46, 86.83)	61.16	(49.09, 69.06)	48.66	(35.19, 61.84)
Left colon	434	87.00	(83.09, 90.06)	69.61	(62.99, 75.26)	60.00	(51.38, 67.68)
Colon-non specified	160	76.84	(69.98, 82.33)	60.85	(52.00, 68.56)	50.44	(41.94, 59.81)
Rectum	234	82.94	(76.89, 87.53)	48.07	(29.00, 64.83)	20.61	(1.82, 53.63)
Tumor grade^*^							
Well differentiated	33	85.91	(66.52, 94,44)	55.75	(26.94, 77.08)	37.17	(12.44, 62.51)
Moderately differentiated	774	87.48	(84.71, 89.78)	69.73	(64.71, 74.18)	57.51	(50.85, 63.59)
Poorly differentiated/Anaplastic	67	62.48	(48.36, 73.75)	43.64	(26.65, 59.47)	–	–
Unknown	138	66.22	(56.6, 74.16)	49.64	(38.16, 61.11)	34.64	(19.75, 51.00)
Stage at diagnosis^*^							
Localized	227	93.67	(89.33, 96.29)	86.12	(77.55, 91.59)	79.85	(68.31, 87.56)
Regional	441	92.66	(89.46, 94.92)	77.92	(83.54, 71.19)	63.25	(53.11, 71.78)
Distant metastasis	338	63.29	(57.36, 68.63)	44.33	(37.65, 51.11)	20.31	(12.96, 28.82)
Unknown	36	69.44	(43.89, 85.60)	38.88	(14.67, 62.82)	–	–
**Surgery**							
Yes	426	92.63	(89.62, 94.79)	75.98	(71.24, 80.77)	62.78	(55.23, 69.42)
No	586	74.25	(69.36, 78.51)	53.44	(46.23, 61.12)	40.09	(31.35, 49.62)
**Chemotherapy**							
Yes	411	90.74	(87.39, 93.23)	67.94	(61.71, 73.38)	52.92	(45.19, 60.06)
No	601	77.17	(73.15, 81.67)	63.69	(57.38, 69.33)	55.08	(46.35, 62.98)
**Radiotherapy**							
Yes	95	87.81	(79.04, 93.06)	56.83	(33.31, 74.79)	38.97	(15.54, 62.66)
No	917	82.31	(79.45, 84.81)	64.83	(61.37, 68.92)	54.04	(45.39, 57.34)

* Indicate no pateints alive to calcuate survival rate for survival year period

* Indicate no pateints alive to calcuate survival rate for survival year period

Table 4 shows the results of univariate and multivariate cox regression analyses. Patients diagnoses at younger age (<=40) and older age (> 70 years), patients presented with advanced stages (regional or distant metastasis), and patients have not treated with surgery and perioperative chemotherapy were all at increased risk of CRC mortality compared to their counterparts. Lastly, as displayed in Tables 4, and 5, demonstrate that 5-year CRC survival among SEER population is higher than those in MNG-HA considerably, except for metastatic 5-year CRC where MNG-HA shows better survival.

**Table 4:** Univariate and multivariate analysis of CRC-specific mortality with in 1,3 and 5 years since diagnosis during (2009–2017) ^a^

Characteristics	Total	Univariate			Multivariate
	N	^**b**^aHR	95% CI	aHR	95% CI
**Diagnosis period**					
2009–2011	288	–	–	–	
2012–2014	395	0.99	(0.74,1.31)	0.91	(0.67, 1.24)
2015–2017	329	0.96	(0.69,1.34)	0.76	(0.52, 1.12)
**Age**					
< =40	40	**1.87**	**(1.09,3.47)**	1.72	(0.49, 6.07)
41–50	68	1.03	(0.58,1.54)	0.73	(0.25, 2.13)
51–60	186	1.02	(0.67,1.54)	0.79	(0.39, 1.59)
61–70	288	–	–	–	–
71–80	237	**1.53**	**(1.09.3.5)**	1.29	(0.72, 2.35)
> =81	193	**2.51**	**(1.8, 3.5)**	1.36	(0.65, 2.84)
**Gender**					
Female	430	1.2	(0.92,1.48)	1.002	(0.77, 1.31)
Male	582	–	–		
**Marital status**					
Single	55	0.91	(0.51,1.63)	0.86	(0.45, 1.61)
Married	748	–	–	–	–
Widowed/divorced	57	1.41	(0.86,2.31)	1.04	(0.6, 1.81)
Unknown	142	0.91	(.068,1.43)	0.92	(0.62, 1.36)
**Nationality**					
Non-Saudi	76	0.66	(0.37,1.18)	0.65	(0.35, 1.19)
Saudi	936	–	–	–	–
**Tumor Site**					
Right colon	184	–	–	–	–
Left colon	434	0.77	(0.54,1.11)	0.72	(0.49, 1.04)
Colon-non specified	160	1.12	(0.76, 1.62)	0.88	(0.58, 1.34)
Rectum	234	1.01	(0.68, 1.51)	0.98	(0.63, 1.54)
**Tumor grade**					
Well differentiated	33	–	–	–	–
Moderately differentiated	774	0.73	(0.38, 1.37)	0.76	(0.38, 1.49)
(Poorly differentiated/Anaplastic))	67	1.98	(0.95, 4.11)	1.52	(0.69, 3.31)
Unknown	138	1.7	(0.86, 3.33)	1.08	(0.51, 2.75)
**Stage at diagnosis**					
Localized	186	–	–	–	–
Regional	362	**1.89**	**(1.17,3.04)**	**2.51**	**(2.51, 4.13)**
Distant metastasis	225	**9.01**	**(5.79,14.71)**	**11.43**	**(7.04, 18.55)**
Unknown	76	**5.51**	**(2.71,11.49)**	**4.87**	**(2.28, 10.37)**
**Basis of diagnosis**					
Histology of primary	972	–	–	–	–
Histology of metastases	28	4.89	(2.98, 8.04)	1.67	(0.92, 3.04)
Others	12	6.42	(3.45,11.78)	7.17	(0.92, 3.04)
**Morphology**					
Adenocarcinoma (AC), NOS	854	–	–	–	–
Mucinous AC	63	1.07	(0.64,1.78)	0.78	(0.45, 1.36)
Mucin-producing AC	12	1.62	(0.66,3.93)	1.58	(0.63, 3.94)
Signet ring cell carcinoma	14	5.07	(2.59,9.91)	1.65	(0.75, 3.61)
AC in villous/tubuvillous adenoma	16	0.071	(0.22,2.21)	0.96	(0.30, 3.08)
Others	53	1.68	(1.08,2.61)	0.62	(0.33, 1.21)
**Surgery**					
Yes	426	–	–	–	–
No	586	**2.48**	**(1.92,3.19)**	**1.36**	**(1.007,1.83)**
**Chemotherapy**					
Yes	411	–	–	–	–
No	601	**1.39**	**(1.07,1.74)**	**1.65**	**(1.17, 2.33)**
**Radiotherapy**					
Yes	95	–	–	–	–
No	917	1.26	(0.82, 1.93)	0.79	(0.49, 1.31)

^a^ Data represent Saudi patients registered in the MNG-HA hospitals system between January 1, 2009, and December 31, 2017

^b^ aHR: Adjusted hazard ratio. Adjusted for all variables in Table 2

Bolded aHR indicates significant *p*-value

**Table 5:** Five-year Colorectal Cancer Survival by Stage at Diagnosis in MNG-HA Compared to US SEER (2009–2017)

	All stages		Localized		Regional		Distance	
	Survival (%)	95% CI	Survival (%)	95% CI	Survival (%)	95% CI	Survival (%)	95% CI
**Male**								
US SEER	65.1	(64.8,65.3)	88.6	(88.3,88.8)	69.9	(69.4,70.4)	13.0	(12.5,13.4)
MNG-HA	53.38	(45.32,67.78)	81.57	(58.51,87.41)	57.89	(43.44,69.89)	19.08	(8.00,33.74)
**Female**								
US SEER	66.5	(66.3,66.8)	90.0	(89.7,90.3)	71.4	(71.0,71.8)	15.2	(14.7,15.7)
MNG-HA	51.14	(43.01,58.68)	–	–	66.62	(51.08,78.22)	21.96	(12.64,32.93)

US SEER (2003–2013) MNG-HA (2009–2017)

## Discussion

As CRC is becoming a public health burden in Saudi Arabia, evinced by lack of screening and increased incidence and mortality [4], it is imperative to elucidate changes in survivorship and shed lights on determinants of poor survivals. While prior studies have investigated survival among Saudi population [9, 10, 20], the determinants of survivals while accounting for potential covariates such as period of diagnosis, case mix, marital status, treatment, and other tumor characteristics has yet to be demonstrated. Additionally, given the poor 5-year survival among Saudi population relevant to other population (e.g., SEER), reporting survival at duration earlier than 5 years is crucial. In this retrospective cohort study, findings showed that there is no significant improvement in CRC-specific survival among MNG-HA population during past decade and found that the 1-, and 3-, survival were 84.08, and 65.16%, respectively.

One of the major findings of this study was that almost two third of cases had relatively late stage at diagnosis (regional/distant). Considering that stage at diagnosis is the most prognostic factor for CRC outcome, it is alarming to see the increasing trends of regional and distant metastasis CRC over time, the proportion with stage (regional/distant) disease increased from 74.02 to 81.76%. The increase in late cancer stages is likely to be as a result of improvements in imaging and diagnostic methods, resulting in a significant shift in stages over the years 2009–2017 in Saudi Arabia. Another possibility is lack of national data on the incidence of adenomatous polyps or the age groups in which the incidence surges, and absence of studies that evaluate effectiveness of different CRC screening test methodologies in Saudi Arabia [3]. While disparities in CRC survival in the US is partially driven by differences in SES and access to care, the disparities present in our study suggest otherwise. For example, given that the entire population at MNG-HA has equitable access to care, late stage at diagnosis (31.79% reported here vs 22% in the US) in our population is primarily driven by lack of screening and potentially high prevalence of the Kristen Rat Sarcoma (KRAS) mutations in colorectal cancer, which is an indicator for poor effectiveness of certain treatments [21]. It is possible though some proportion of late-stage cases could have been developed due to delayed appointment, which should be investigated in future studies.

In multivariate analysis, we found that patients diagnosed at late cancer stage were more likely to die of their tumor compared to the early stages. Same has been found among SEER population and other studies across Europe [12, 22–26]. The prognosis of distant metastasis CRC is very poor since diagnosis at this stage is associated with high morbidity and mortality regardless of patient’s characteristics. Nonetheless, the only established method that has been associated with downstaging at the population-level is CRC screening [27], which is currently weak in Saudi Arabia despite recent calls for action [4, 9, 28]; hence the increasing percentage of distant metastasis tumors.

Over the study period, more than 80% of NGHA CRC patients have been treated with surgical resection of the primary tumor which has been the traditional approach to manage late CRC stage, or/and chemotherapy. In multivariate analysis, we found that patients treated with surgery or chemotherapy, have significant lower risk of death than those who did not use treatment. Similar results have previously been described [29–33]. Prolonged survival outcomes have been shown among CRC patients who were treated with both surgery and chemotherapy [29, 31]. Additionally, a meta-analysis conducted in 2015 emphasized on the survival benefits of chemotherapy alone, showing that oxaliplatin and capecitabine or infusional/bolus 5-fluorouracil-based chemotherapy plus bevacizumab resulted in prolonged survival [31].

One of the strengths of the current study is the use of cancer registry of unique military population diagnosed with CRC and has never been studied. While the majority of MNG-HA are military personal, resembling the VA in the US, some are dependents who are white collar or students. Current study has nonetheless several limitations that should be noted. First, the findings should be generalizable to MNG-HA population or other similar population. Second, the reported CRC survival should be interpreted within the purview of CRC incidence and mortality rates in Saudi Arabia [34, 35]. Given the increasing rates of CRC incidence during the study period, and increased mortality [4, 8]; although there is lack of mortality data, our findings suggest an increased disease burden. This lack of progress could be ameliorated through reduction in CRC risk factors, increased population-based screening and more effective stage-based treatment. Third, no treatment information was used in this study.

## Conclusion

The current study characterized for the first time the CRC survival profile among MNG-HA population, revealing disproportionately poor survival compared to the US SEER data. Despite advancement in the Saudi healthcare, the fact that 5-year survival (52%) is markedly lower than those reported among SEER population (65%) and even lower than SEER’s 10-year survival (58%) presents a future challenge. To increase survival, it is imperative to adopt evident strategies at the population level that lead to downstaging such as CRC screening. The US National Polyp Study, for instance, found that polypectomy result in 53% reduction in CRC mortality and up to 76% reduction in the incidence of CRC.

## Data Availability

The data are available from the Oncology Department, but restrictions apply to the availability of these data due to sensitive identifier that have been used in this study, which were used under license for the current study, and so are not publicly available. For further assistance please contact the corresponding author.
